# Aspartic proteinase napsin is a useful marker for diagnosis of primary lung adenocarcinoma

**DOI:** 10.1038/sj.bjc.6600879

**Published:** 2003-04-15

**Authors:** T Ueno, S Linder, G Elmberger

**Affiliations:** 1 Department of Oncology-Pathology, Cancer Center Karolinska, Karolinska Institute and Hospital, SE-171 76 Stockholm, Sweden

**Keywords:** napsin, adenocarcinoma, lung cancer, TTF-1, surfactant protein

## Abstract

Napsin A is an aspartic proteinase expressed in lung and kidney. We have reported that napsin A is expressed in type II pneumocytes and in adenocarcinomas of the lung. The expression of napsin was examined in 118 lung tissues including 16 metastases by *in situ* hybridisation. Napsin was expressed in the tumour cell compartment in 33 of 39 adenocarcinomas (84.6%), in two of 11 large cell carcinomas and in one lung metastasis of a renal cell carcinoma. Expression of napsin was found to be associated with a high degree of differentiation in adenocarcinoma. Immunohistochemistry was performed for three proteins currently used as markers for lung adenocarcinoma : surfactant protein-A, surfactant protein-B and thyroid transcription factor-1. Thyroid transcription factor-1 showed the same sensitivity (84.6%) as napsin for adenocarcinoma, whereas surfactant protein-A and surfactant protein-B showed lower sensitivities. Among these markers, napsin showed the highest specificity (94.3%) for adenocarcinoma in nonsmall cell lung carcinoma. We conclude that napsin is a promising marker for the diagnosis of primary lung adenocarcinoma.

Lung cancer is the leading cause of cancer mortality in the world and one of the top incidence of cancers in Europe and the US (Black *et al*, 1997; Wingo *et al*, 1999). Lung cancer has a poor prognosis and even for patients with operable disease, the 5-year survival rate is 14% in the US (Landis *et al*, 1998). Nonsmall cell lung cancer (NSCLC), essentially consisting of adenocarcinoma, squamous cell carcinoma (epidermoid carcinoma) and large cell carcinoma, accounts for approximately 80% of all lung cancers (Travis *et al*, 1995). The incidence of pulmonary adenocarcinoma has been increasing and adenocarcinoma is now the most common histologic subtype in the US (Charloux *et al*, 1997; Landis *et al*, 1998).

The lung is also a common site for metastases from tumours growing at other sites. From a clinical point of view, it is important to distinguish between primary lung adenocarcinoma and metastatic adenocarcinoma in the lung since treatment protocols differ depending on the origin. Primary lung adenocarcinoma develops from type II pneumocytes and from bronchiolar nonciliated secretory cells (Clara cells). Surfactant apoproteins, including surfactant protein-A (Sp-A) and surfactant protein-B (Sp-B) are used as markers for lung adenocarcinoma and thyroid transcription factor-1 (TTF-1) is used as a marker for lung adenocarcinoma, large cell carcinoma, small cell carcinoma and thyroid carcinoma (Kaufmann and Dietel, 2000b).

Recently, a new aspartic proteinase, napsin A, was cloned and shown to be expressed in lung and kidney (Tatnell *et al*, 1998). Our group and another group have shown that napsin A is identical to the protein spots TAO1/TAO2 detected by two-dimensional gel electrophoresis of lung adenocarcinoma, and that napsin A is expressed in type II pneumocytes and lung adenocarcinomas (Chuman *et al*, 1999; Hirano *et al*, 2000). However, the detailed expression patterns of napsin A in association with other markers in lung cancer remain to be clarified. In this study, we examined napsin expression by *in situ* hybridisation and performed immunohistochemistry for Sp-A, Sp-B and TTF-1 using 118 lung tumour specimens to investigate the expression of napsin and other markers in lung cancer.

## MATERIALS AND METHODS

### Tissues

One hundred and eighteen lung tumour tissues, from patients treated in the Karolinska Hospital from 1994 to 1999, were selected for this study. The samples included 39 adenocarcinomas including seven bronchioloalveolar carcinomas (BAC), 11 large cell carcinomas, 31 squamous cell carcinomas, 15 small cell carcinomas, six carcinoids and 16 metastasised tumours in the lung. The origins of the metastasised tumours included four melanomas, one renal cell carcinoma, one gastric carcinoma, one colon carcinoma, one salivary gland cancer, one prostate cancer, one midline germ cell embryonic carcinoma, one basal cell carcinoma and five sarcomas. The tissues were surgically resected except small cell carcinoma and metastasised tumour tissues that were obtained by biopsies. Forty-three were from female patients and 75 were from male patients. The average age of the patients was 62.4 years (range: 16–85). The study was approved by the local institutional review board. The tumours were classified according to the WHO histological typing of lung tumours (second edition).

### *In situ* hybridisation

Formalin-fixed, paraffin-embedded tumour sections were deparaffinised with xylene, treated with proteinase K (1 *μ*g ml^−1^, 37°C, 30 min), transferred to 0.1 M triethanolamine buffer (5 min) and treated with triethanolamine containing 0.25% acetic anhydride for 10 min. Sections were washed in 2 × SSC, dehydrated and allowed to air-dry. After overnight hybridisation (2 × SSC, 50% formamide, 10% dextran sulphate, 55°C) with an ^35^S-labelled RNA probe (1.16 × 10^5^ c.p.m. *μ*l^−1^), sections were washed (the most stringent step being 0.1 × SSC, 15 min at 60°C) and treated with RNAse A (20 *μ*g ml^−1^, 37°C, 30 min). Finally, the slides were dehydrated, air-dried, dipped in Kodak NTB emulsion, exposed for 6–21 days at 4°C, developed and counter-stained with haematoxylin–eosin. The probes were made using T3 (antisense) and T7 (sense) RNA polymerase (Promega, Madison, WI) from a pCMS-EGFP vector (BD Biosciences Clontech, Palo Alto, CA) containing an *Nhe*I/*Eco*RI napsin A full-length cDNA. Both sense and antisense probes were hybridised to all sections.

### Immunohistochemistry

Formalin-fixed, paraffin-embedded lung tissues were subjected to immunohistochemistry using an anti-Sp-A polyclonal antibody (Chemicon International Inc., Tamacula, CA), an anti-Sp-B monoclonal antibody (personal gift from Dr Y Suzuki, Japan) and an anti-TTF-1 monoclonal antibody (DAKO, Copenhagen, Denmark). The sections were stained using the Ventana NexES Staining system (Ventana Medical System, Tucson, AZ, USA). Briefly, 4-*μ*m sections were incubated with the primary antibody for 30 min at 37°C and with a secondary biotinylated antibody for 10 min.

The stained slides were analysed microscopically by three observers in consensus and the results were expressed on a plus–minus scale on the basis of the proportion of tumour cells stained: 0–10%, −; 10–50%, +; 50–100%, ++. Only staining with apical membranous or cytoplasmic staining pattern was accepted as positive when stained with Sp-A/Sp-B. For TTF-1, only nuclear staining pattern was accepted as positive although our series contained a few tumours showing distinct specific cytoplasmic staining pattern without background. The sensitivity of each protein for primary adenocarcinoma and the specificity of each protein for adenocarcinoma among NSCLC were calculated as follows:

Sensitivity=the number of adenocarcinomas expressing each marker/the total number of adenocarcinomas (39),

Specificity=the number of adenocarcinomas expressing each marker/the number of NSCLC (adenocarcinoma, large cell carcinoma and squamous cell carcinoma) expressing each marker.

## RESULTS

**Figure 1:**
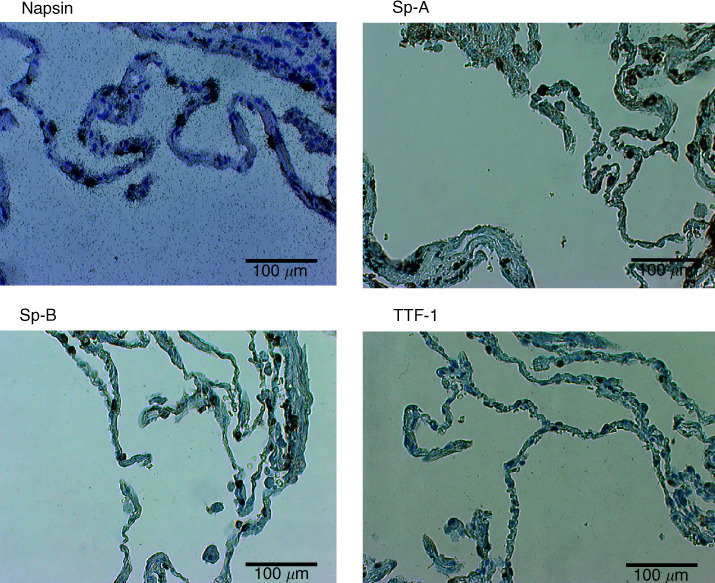
Expression of napsin, Sp-A, Sp-B and TTF-1 in type II pneumocytes.

Napsin expression was studied by *in situ* hybridisation in sections containing normal lung tissues. Napsin expression was observed in alveolar type II pneumocytes (Figure 1

), but not in other types of cells including bronchiolar epithelium and bronchial epithelium. Hybridisation with a sense probe resulted in a diffuse background and did not show any specific localisation (data not shown).

Expression of Sp-A, Sp-B and TTF-1 was examined by immunohistochemistry. Staining was observed in alveolar type II pneumocytes. Sp-A showed a membranous or cytoplasmic staining pattern and Sp-B showed cytoplasmic staining, whereas TTF-1 showed a nuclear staining pattern (Figure 1). All four markers were expressed in reactive type II pneumocyte hyperplasia lesions (data not shown).

**Figure 2:**
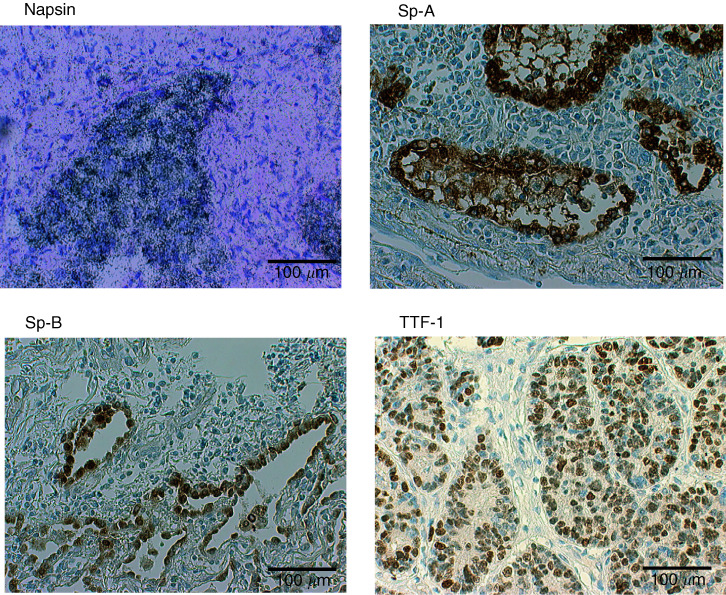
Expression of napsin, Sp-A, Sp-B and TTF-1 in lung adenocarcinoma.

Napsin expression was examined in 118 lung tumour tissues. Only expression by tumour cells was considered. Thirty-three of 39 adenocarcinomas (84.6%) expressed napsin mRNA (Figure 2

) and two of 11 large cell carcinomas showed napsin mRNA expression (Table 1

**Table 1:** Expression of markers in lung tumours

	**Napsin**	**Sp-A**	**Sp-B**	**TTF-1**	**Total**
Adenocarcinoma	33	24	24	33	39
Large cell carcinoma	2	3	1	6	11
Squamous cell carcinoma	0	4	5	4	31
Small cell carcinoma	0	0	1	12	15
Carcinoid	0	0	0	3	6
Metastatic tumour^a^	1 (renal cell carcinoma)	1 (salivary gland)	2 (salivary gland, midline germ cell embr. carcinoma)	0	16
					
Sensitivity^b^ (%)	84.6	61.5	61.5	84.6	
Specificity^b^ (%)	94.3	77.4	80.0	76.7	

a Brackets show the origins of metastatic tumours.

b Sensitivity and specificity for adenocarcinoma among nonsmall cell lung cancer (adenocarcinoma, large cell carcinoma and squamous cell carcinoma).

). One lung metastasis of renal cell carcinoma origin expressed napsin mRNA, consistent with the previously described expression of napsin A in epithelial cells of renal tubules (Schauer-Vukasinovic *et al*, 1999). None of the other types of tumours expressed napsin mRNA.

Sp-A, Sp-B and TTF-1 were expressed in adenocarcinomas (Figure 2) but the expression was also observed in other types of lung tumours (Table 1). In particular, TTF-1 expression was observed in a large proportion (80%) of small cell carcinomas, in agreement with previous reports (Fabbro *et al*, 1996; Kaufmann and Dietel, 2000a). The sensitivity and specificity of each marker for the diagnosis of adenocarcinoma among NSCLC were calculated. Napsin and TTF-1 showed the highest sensitivity (84.6%), and napsin had the highest specificity (94.3%). Two adenocarcinomas were positive for napsin but negative for TTF-1. Combining napsin and TTF-1 resulted in a higher sensitivity for adenocarcinoma (89.7% (35 out of 39)) whereas the specificity was the same as TTF-1 alone (76.7%).

**Figure 3:**
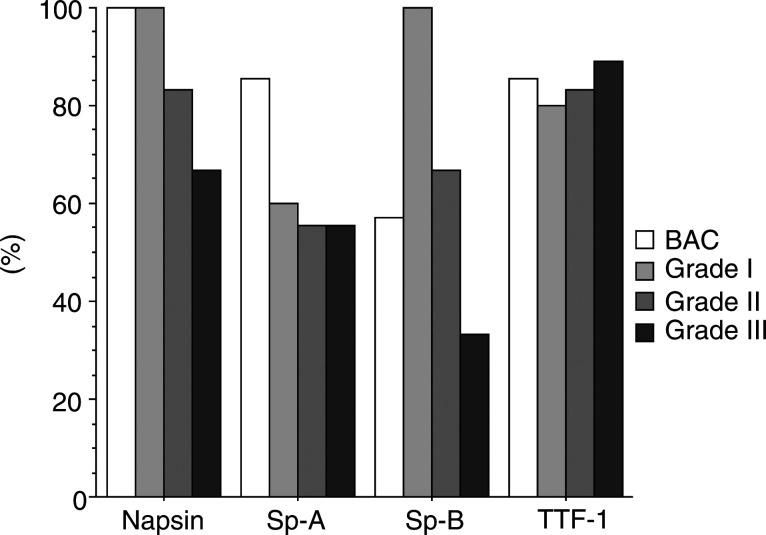
Expression of each marker in relation to differentiation grade of lung adenocarcinoma. Napsin showed an association with the differentiation grade of adenocarcinoma. A similar association was observed for Sp-A.

The association between the expression of each marker and differentiation grade of the adenocarcinoma is presented in Figure 3

. Expression of napsin and Sp-A were associated with a high degree of tumour differentiation.

## DISCUSSION

Napsin is found in two isoforms, napsin A and B, with highly homologous nucleotide sequences (91.2%) (Tatnell *et al*, 1998). Napsin A appears to be a functional proteinase, predominantly expressed in lung and kidney. Napsin B is transcribed exclusively in cells related to the immune system and lacks an in-frame stop codon and is believed to be a pseudogene (Tatnell *et al*, 1998). In the present study, napsin expression was examined by *in situ* hybridisation of tissue sections. Expression was specifically assessed in type II cells and the tumour cells in lung carcinoma tissue. Hybridisation signals from other cell types (e.g. lymphocytes) were not considered to avoid possible scoring of napsin B expression.

We demonstrated napsin expression in type II pneumocytes, which are identified by approximately cuboid shape, large and roundish nucleus and cytoplasmic staining for Sp-A (Ten Have-Opbroek *et al*, 1997). Type II pneumocytes are thought to be progenitor cells of normal and neoplastic epithelial cells during the repair of injury and during carcinogenesis (Adamson and Bowden, 1974; Mizutani *et al*, 1988; Linnoila *et al*, 1992). Napsin mRNA was expressed in not only normal type II pneumocytes, but also reactive type II pneumocyte hyperplasia and adenocarcinoma, suggesting that napsin may be used as a novel type II lineage marker.

Three different markers, Sp-A, Sp-B and TTF-1, are currently used for the differential diagnosis of primary lung adenocarcinoma. TTF-1 appears to be the most sensitive of these markers (Kaufmann and Dietel, 2000b). In agreement with that study, we found a higher sensitivity of TTF-1 compared to Sp-A and Sp-B. Napsin showed the same sensitivity as TTF-1. However, the specificity of napsin to adenocarcinoma was the highest among the markers examined. Napsin mRNA was expressed in primary lung adenocarcinomas and in some large cell carcinomas, but not in other types of primary lung cancers. This is in distinction from TTF-1, which is also expressed in a large proportion of small cell carcinomas (Table 1) (Fabbro *et al*, 1996; Kaufmann and Dietel, 2000a). Napsin expression was examined in a restricted number of lung metastases. Expression was observed in one metastatic tumour, from a renal cell carcinoma. This observation is consistent with previous findings of napsin expression in renal tubules (Schauer-Vukasinovic *et al*, 1999). Clearly, however, a large number of metastases from different primary tumours have to be examined before a firm conclusion as to the value of napsin as a marker for the differential diagnosis between primary and metastatic lung carcinoma can be made. The restricted tissue distribution of napsin suggests that this proteinase may be a useful marker.

Some aspartic proteinases are involved in cancer progression. Overexpression of cathepsin D in transformed cells increases a metastatic potential in mice (Garcia *et al*, 1990). High cytosolic cathepsin D levels are associated with poor prognosis in primary breast cancer (Foekens *et al*, 1999). Although we did not find an association between napsin expression and patients' prognosis in this study (data not shown), a correlation between napsin expression and the differentiation grade of adenocarcinoma was observed in accordance with previous reports (Hirano *et al*, 1997,^2000^). The high positive rate (more than 80%) of napsin in lung adenocarcinoma and its close association with the differentiation grade may suggest a role of napsin in cancer development or differentiation.

In conclusion, we showed that napsin was expressed in a large proportion of primary lung adenocarcinoma and showed its association with the differentiation grade. The results suggest that napsin is a promising marker for differential diagnosis of adenocarcinoma in the lung.
